# The Journey of Recovery and Empowerment Embraced by Nature — Clients’ Perspectives on Nature-Based Rehabilitation in Relation to the Role of the Natural Environment

**DOI:** 10.3390/ijerph110707094

**Published:** 2014-07-14

**Authors:** Anna María Pálsdóttir, Dennis Persson, Birgitta Persson, Patrik Grahn

**Affiliations:** 1 Department of Work Science, Business Economics and Environmental Psychology, Swedish University of Agricultural Sciences, P.O. Box 88, SE-230 53 Alnarp, Sweden; E-Mails: birgitta.persson@slu.se (B.P.); patrik.grahn@slu.se (P.G.); 2 Department of Health Science Center, Occupational Therapy and Occupational Science, Lund University, P.O. Box 117, SE-221 00 Lund, Sweden; E-Mail: dennis.persson@med.lu.se

**Keywords:** supportive environment, horticulture therapy, stress restoration, therapeutic landscape, evidence-based health design, social quietness

## Abstract

This paper presents findings from real life situations, a longitudinal single case study on the role of natural environments in nature-based rehabilitation (NBR) for individuals with stress-related mental disorders, at the Alnarp Rehabilitation Garden in Sweden. A sample of 43 former clients voluntarily participated in semi-structured interview, and the data were analyzed according to interpretative phenomenological analysis (IPA). Three main superordinate themes were identified as the three phases of NBR—*Prelude*, *Recuperating* and *Empowerment*—explaining and illuminating the role of the natural environments in each phase. An explanatory model of NBR in this context is presented including the three phases of NBR, *IRP* supportive occupations and a pyramid of supporting environments. A new component of supportive environments was identified and herby named, *Social quietness*, an important component facilitating personal and intimate engagement with the natural environments.

## 1. Introduction

Mental health problems are estimated to be among the major global contributors to work disabilities [1,2]. In Sweden, sick leave due to mental health problems has increased in recent years [3,4,5], and according to a Swedish Social Insurance Agency report, the most common cause of sickness absence from work is stress-related mental disorders [3]. It is well known that chronic stress exposure, mostly studied as work-related stress, can result in clinical symptoms and mental complaints, often referred to as stress-related mental disorders [6,7]. The term “stress-related mental disorders” is most commonly used to describe mental disorders mainly caused by psychosocial stress, such as fatigue, burnout, exhaustion, depression, anxiety or adjustment disorder. The burnout concept, which has been widely used in stress research, is commonly defined as a mental condition that has developed as a result of continuous stress exposure particularly related to psychosocial factors at work [8]. However, burnout is mostly used to study working populations and is commonly not used in clinical practice. The clinical diagnosis “Exhaustion disorder” (ED) was proposed by the National Board of Health and Welfare in Sweden [9] for use in clinical practice to define patients with exhaustion that has developed as a consequence of identifiable stressor(s) that have been present for at least six months. ED was established to improve diagnostics in cases of stress-related exhaustion and was assigned the code F43.8 of ICD-10 [9,10,11]. The symptoms of ED and burnout are closely related, and it has been previously shown that the majority of patients fulfilling the diagnostic criteria for ED can also be described as being burned-out [12,13]. The core symptoms of patients with ED are severe tiredness and exhaustion, with impaired executive functions as well as mental and physical impairments [9,12]. The recovery process [14] for individuals with ED has been described as vulnerable and can take months or even years [15,16]. It is recognized that individuals reporting mental health problems such as depression and exhaustion that have developed as a consequence of high stress exposure are in great need of rest, especially before taking an active part in the rehabilitation [16].

There is an urgent need to find rehabilitation alternatives that successfully support recovery process [17,18] for individuals with ED. Rehabilitation is the process practitioner use for facilitating recovery [19] and recovery refers to the lived experience (the process) the patient undergoes [14]. Recovery is regarded as highly individual and unique process [20,21]. Today, the recommended rehabilitation for individuals with ED include multimodal rehabilitation addressing issues like occupational balance in everyday life, stress management, psychodynamic therapy (PDT) or cognitive behavioural therapy (CBT) in-group or individually, and vocational therapy [22]. However, the environments where the rehabilitation takes place are not considered in these recommendations. It has been argued that the environment is an important part of the rehabilitation and that specifically natural environments can support the recovery process for individuals with stress-related illnesses [23,24]. Natural environments can support therapeutic processes and act as a therapeutic partner in various types of therapeutic interventions [25,26].

There is increasing scientific evidence that Nature can be a positive resource for relieving symptoms of stress and improving mental recovery [27,28,29,30]. In order to investigate nature’s positive effects on individuals with stress-related mental disorders, a nature-based rehabilitation (NBR) environment was developed at the Alnarp campus of the Swedish University of Agricultural Sciences. The NBR is performed in a specially designed garden, with a selected transdisciplinary treatment team and a specially developed NBR adapted to treat stress-related mental disorders [24,30,31,32].

In a recent study [33] the results from the NBR at Alnarp rehabilitation garden were compared with a matched reference population receiving: treatment as usual. A significant reduction in healthcare consumption was noted among participants in the NBR compared with the reference population. The main changes were a reduction in outpatient visits to primary healthcare and a reduction in inpatient psychiatric care. Newly published results [32] show that levels of function and return to work rate increased after NBR at the Alnarp Rehabilitation Garden and that this was significantly associated with changes in the participants’ everyday lifestyles.

Other NBR for people with stress-related mental disorders has also been developed [34,35,36,37,38,39,40] and the evidence supporting NBR is accumulating, e.g., a decrease in the symptoms of the disease [32,33,37,39]. None of the above mentioned studies handle the physical environment where the NBR takes place. There is a need to identify and describe specific aspects of the natural environments that may support health processes [41,42].

The purpose of the present study was to explore and illustrate how participants with stress-related mental disorders participating in nature-based rehabilitation experience and describe their rehabilitation process in relation to the role of the natural environments at the Alnarp Rehabilitation Garden.

## 2. Methods

This study was designed as longitudinal single case study [43,44] using semi-structured interviews [44,45] and interpretative phenomenological analysis (IPA) [46,47].

### 2.1. Single-Case Study

A single-case study can be motivated if the case can be defined as something unique or extreme and is thus considered an extraordinary and well-defined event [48]. The Alnarp Rehabilitation Garden is a unique phenomenon in its use of a professional healthcare rehabilitation team and a specially designed outdoor environment (the garden). It is a living laboratory for studying, in a real-life context, the interaction between individuals and the environment in nature-based rehabilitation. The whole NBR concept is the subject of SET, in which the individual is the central figure and receives support from the social and physical environment through different occupations. It is a critical case for testing well-formulated theories [44]. The NBR at the Alnarp Rehabilitation Garden is defined as a supportive environment consisting of the multimodal and transdisciplinary rehabilitation team, the group of eight participants, the individual him/herself, occupations, and the specially designed garden (the natural environment). The aim of the study was to increase the in-depth understanding of supportive outdoor environments, especially the role of nature in the rehabilitation process and the essential qualities by which nature can affect health outcomes. In the current work, both descriptive and explanatory approaches were applied to investigate the case in question.

### 2.2. The Study Context

**Figure 1:**
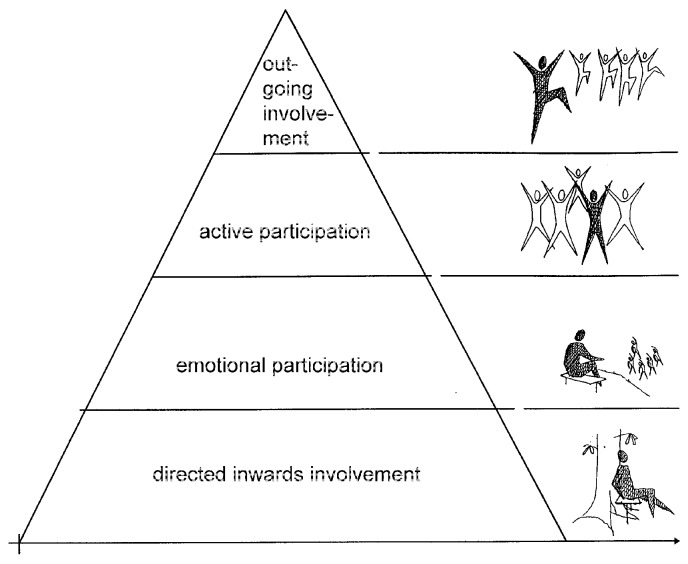
The supportive environment theory (SET) pyramid. The pyramid is divided into four levels of executive functions, the lower part symbolizing low capacity of executive functions and characterized by inward involvement and a high need for a supportive environment. Meanwhile, the higher levels symbolize higher capacity of executive functions and are characterized by active or outgoing involvement and less need of a supportive environment.

The study was carried out at the Alnarp Rehabilitation Garden [23,24]. The two-hectare rehabilitation garden was designed according to theories on nature’s restorative effects [29,49,50] and supportive environments (SET) [24]. SET is illustrated with a pyramid (see Figure 1) in which the social and physical environments are related to a person’s executive functions [51]. The pyramid is divided into four levels of executive functions, the lower part symbolizing low capacity of executive functions and characterized by inward involvement and a high need for a supportive environment. Meanwhile, the higher levels symbolize higher capacity of executive functions and are characterized by active or outgoing involvement and less need of a supportive environment [24].

The garden contains places for work as well as for rest and contemplation, and is divided into two major areas: the *Nature Area*, an informal nature garden; and the *Cultivation and Gardening Area*, including formal gardens and rooms for horticulture and other garden work [24]. It is further subdivided into different garden rooms for supporting and embracing the clients’ rehabilitation process [23,31]. The garden is fenced, with a large gateway as its main entrance. The area is closed to visitors during the NBR takes place [52].

The NBR was designed as 12 weeks’ rehabilitation and was performed as group therapy consisting of a maximum of eight people in each group, and as one group ended another begun. All participants in this study completed the 12 weeks of NBR. A transdisciplinary team, integrating four major therapy forms, supported the NBR: occupational therapy, physiotherapy in the form of Rosen therapy, psychotherapy and horticultural therapy. The aim of the NBR [24] was to enhance a salutogenic and curative process to reinforce each individual’s physical and mental capacity through connecting to their inner self with firm support from natural environments. For a more detailed description of the NBR, see Grahn *et al.* [24], Pálsdóttir *et al.* [32] and Lavesson [53].

### 2.3. Participants

The study sample consisted of 43 former clients (35 female and eight male), all of whom had participated in a 12-week NBR. The inclusion criteria for participating in the NBR were (i) on long-term sick leave; and (ii) one of the following International Classification of Diseases (ICD-10) as the primary diagnosis: psychiatric diagnosis of adjustment disorder and reaction to severe stress (ICD-F43), or depression (ICD-F32.0, F32.1). The exclusion criteria were known drug and alcohol abuse. All participants were Swedish residents, and their age range was 25–62 years (mean 45.5 years). Their professions included various academic and practitioner positions.

### 2.4. Data Collection

This single-case study concerned the location and the intervention as a current whole, in this study defined as nature-based rehabilitation. A semi-structured interview approach was chosen, as it can explore and illuminate how clients experience their rehabilitation process in relation to the role of the natural environments in this specific context [45]. A longitudinal approach [44] was chosen to cover the seasonal variation as well as the variation over several years and the study was carried out during the period of four years, in 2007 and from 2009 to 2011. All study participants signed written consent before the interview took place [54]. The first author individually interviewed each client within one month after they ended their rehabilitation, and all interviews were audio-recorded and transcribed verbatim. The approximately hour-long interview was conducted with an informal approach, aided by an interview guide concerning the participant’s experience of nature-based rehabilitation in relation to the role of the natural environments at the Alnarp Rehabilitation Garden. A map of the garden area was used to ensure accuracy, *i.e.*, that the informant and interviewer were discussing the same location in the garden, as well as to generate more discussion. An ethnographic approach was chosen as it allowed the researcher to see and experience *in situ* how the intervention took place [55]. The first author participated in the NBR, first during spring 2010 and then again during spring 2012, observing the intervention as a current whole phenomenon. Prior to the observational study, the participants were informed of its purpose and consensus approval regarding the researcher entering the group was attained [56].

### 2.5. Data Analysis

The transcripts were subjected to IPA, an idiographic and detailed analysis of elements reflecting persons’ experiences of an event or a phenomenon and how they give meaning to it [46,47,57]. IPA entails a hermeneutic approach to interpretation, and does not seek a saturation of themes and is not a theory inspired analysis. As the IPA analysis method is taken in several steps, it allows the researchers to approach the studied phenomenon in depth. It is a transparent analysis method with a straightforward approach that the co-authors can easily follow throughout the analysis process.

In IPA, the researcher’s background and previous knowledge of the phenomenon studied are recognized as resulting in a more deeper understanding of and reflection on the phenomenon studied [46,47]. The analysis focused on the whole recovery process in the same way for each participant and did not look at differences between seasons, gender or age.

In the first step of analysis, each manuscript was read while listening to the recorded interview to ensure that the content of the manuscript reflected what had been said. After this, the first author read each manuscript several times and made notes in the margins, identifying important themes. These notes were compared with the ethnographic field notes written during participation in the NBR. Throughout the whole process of the analysis, the work was discussed with the last author, who also read some of the interviews to establish his own perception of the content and the emerging themes. The themes, including comprehensive extracts supporting each theme, were extracted into a summary document. When the first and last authors had reached agreement on the main themes, subordinate themes, subthemes and dimensions, the findings were discussed within the entire group of four authors, who together agreed on the final version. The final results were revealed to the therapists, experts on the client group that was the subject of this study, for discussion of the trustworthiness of the themes that had emerged. The therapists confirmed that the themes were in accordance with what they encounter in their work.

The regional ethical committee in Lund, Sweden, received a formal application for ethical approval but the study was not considered relevant for evaluation as the participants no longer were in the NBR and could voluntarily participate in the study on their own terms. However, they advised of a written consensus before the participants voluntarily entered the study. The participants could at any point end the interview without further explanations.

## 3. Results

Three superordinate themes emerged: *Prelude*, *Recuperating* and *Empowerment*. The order of superordinate themes and subthemes is in the sequence with which the participants described the development of the process (Table 1). The length of each phase *i.e.*, *Prelude*, *Recuperating* and *Empowerment* was highly individual. The dimensions described are parts of an interwoven process within each phase rather than separate sequences like the themes.

**Table 1:** Three superordinate themes emerged from the data: *Prelude*, *Recuperating* and *Empowerment*. The order of superordinate themes and subthemes is in the sequence with which the participants described the development of the process. The dimensions described are parts of an interwoven process within each phase rather than separate sequences like the themes.

Superordinate Themes	Subthemes	Dimensions
Prelude	*Alliance*	*Establishing contact*
	*Permissiveness*	*Armour off*
Recuperating	*Restoration*	*Being present*
		*Being one with nature*
		*Peace and tranquillity in nature*
	*Awakening & processing*	*Entrusting nature*
		*Inspired by nature*
Empowerment	*Moving on*	*Challenging oneself*

### 3.1. Prelude

Two subthemes emerged: *Alliance* and *Permissiveness*. It was important to safely settle into the new environment before the participants could start taking notice of everything else around them: “*First settle in, feel safe and know the others, then start opening up and breaking patterns.*”

#### 3.1.1. Alliance

*Establishing contact*. The participants explicitly expressed that the entrance through the gate into the garden marked the border between their hazardous everyday life and a place of seclusion and security. The garden was closed to visitors (outsiders) during the time of the intervention, which in turn offered privacy and satisfied the participants’ need to feel secure in these new circumstances. The gate became the symbol of a world of sanctuary: “…*it’s enough for me to just look at that gate, how can I put it, yeah I mean I don’t have to achieve anything here; it’s a sanctuary, it’s a sanctuary, yeah.*”

The whole physical environment was perceived as a coherent whole where no odd pieces, materials or colours disturbed the experience of being in a well-balanced and harmonized wholeness: “*it’s not cluttered so that you get a whole lot of sensory impressions; instead, it’s calm and peaceful somehow.*” The lush garden enhanced the feeling of embracement, and soon became a neutral meeting place where new arrivals could establish contact and a relationship with the environment, the team, the group and themselves: “*As an individual, I can meet with these people and try to create a relationship with them based on nothing more than a garden.*”

A well-structured weekly scheme gave a feeling of professionalism. At the beginning of the NBR, the participants experienced the team as an important factor for making them feel at ease and secure. Here, the participants did not need to use their energy on defense but could instead focus on noticing the presence of the safe and secure atmosphere and then enter the context of NBR: “*When you’ve found security, you can show yourself to the staff, the environment, the group and yourself.*”

The participants felt that being amongst others with mutual experience of what the illnesses has brought about made them a group of equals, had no need for words concerning their condition, and could relax: “*Before I’ve always felt that when I meet people I don’t know and stuff like that I was always tense and… but since everybody’s in the same situation it’s been so… yeah, felt like I’ve been able to let a bit more out.*”

#### 3.1.2. Permissiveness

*Armour off*. Once the alliance was under establishment, the participants gradually noticed what they expressed as permissiveness. As they constantly carried the heavy burden of fulfilling other people’s needs and demands, the new experience of permissiveness became an important step for further engaging in the NBR: “*In order for the healing and recovery to take place, the sense of unconditionality is essential.*”

Many participants explained how the tamed and structured parts of the garden, especially in the beginning, were perceived as demanding and as symbolizing demands for and expectations on achievements. In contrast, the more natural and wild parts of the garden were perceived as less demanding, as nature takes care of itself. The undemanding parts gave a sense of freedom to do nothing. In this phase of the rehabilitation process, participants learned to perceive a state of “just being” as restful and permissive: “*In nature I don’t feel like I have to achieve anything. It’s enough to just ‘be’, and that makes me calm.*”

The garden and horticulture occupations were experienced as undemanding tasks, as the participants could enter and leave these as they pleased and had no responsibility for the work to be completed, or for its outcomes. This was a new and pleasant experience, to just be able to enjoy occupations without the usual approach with a focus on performance and results but rather only for the joy of it: “*We had sown basil and oregano, taken geranium cuttings, and propagated mint. I went there to check on the plants, what had happened since the last time—I thought it was fun.*”

The teams’ non-judgemental attitude was perceived as crucial for the evolving feeling of permissiveness, and was experienced as something different than what occurs in other social interactions. This gave the participants an unexpected source of energy and positive attitudes towards their own potential for recovery. This was described as an attitude they had not felt before in other rehabilitation interventions: “*Yeah, and I think it’s so important to take, I mean to stress that this group has pushed themselves for far too many years. It’s of no help if the authorities then also push; that’s the worst they can do. It’s not that we need to be threatened to do our best; we do that anyway, and have done it our whole life. We needed the possibility to rest instead of being pushed to get better; otherwise nothing would happen.*”

The group of participants had a mutual understanding of the difficulties their illness had caused and could thus drop the social politeness, as everyone understood the lack of strength to keep up appearances. The sense of coherence grew and there was no need for explanation or excuses for their actions, as each person easily recognized her/himself: “*I was afraid to be in a group, but this went away fast because we were all on the same level and you could leave the group without having to explain yourself.*”

### 3.2. Recuperating

The second superordinate theme, *Recuperating*, is characterized by the participants’ mental and physical recovery supported by nature. Two subthemes emerged from this superordinate theme: *Restoration* and *Awakening & processing.* In this phase, the interaction between the participants and nature becomes more personal and intimate: “*After a while I felt like the garden did a great deal; the team was important, while the garden grew ever more important as time went on.*”

#### 3.2.1. Restoration

Three dimensions were distinguished: *Being present, Being one with nature*, and *Peace and tranquility in nature.* This phase describes the way nature offers opportunities for rest and recovery as a prerequisite for moving on: “*Rest first, and then stand up and walk.*”

*Being present*. The horticultural and garden occupations were used to remind the participants to take breaks during their work. During the break, some participants took a walk in the garden while others chose to sit in the garden before returning (or not) to their task. The plants in the garden served to capture the moment; *i.e.*, the team used them for close-up encounters, inviting participants to smell, touch, taste or look at the plants. The participants reported that this helped them stay in the feeling they were experiencing at that moment. After a while they grew accustomed to the slower pace, and frequently stayed in the moment through sensory experiences in the garden: “*I stood still quite a lot. I could sort of be there next to a flower for a really long time and just look at and smell and pet, or how can I put it, feel it.*”

*Being one with nature*. Many of the participants expressed the feeling of a re-attachment to nature, and described an underlying need for nature, the nature humans had originated from. A strong feeling of being one with nature allowed them to get closer to their inner feelings: “*Nature is the soul’s food and drink.*”

Several participants mentioned the negative effects of the urban lifestyle. Before, humans followed nature’s rhythm with a balance of rest and play; but now, because of all the technology there is a faster rhythm, accelerating and pressing people’s limits to the utmost. Nature cannot be hurried; things have their own rhythm—*i.e.*, a natural rhythm, not man-made. This inspired many participants to redefine their rhythm of life. Urbanization has resulted in a lack of proper rest and recovery, which nature offers: “*People’s origins are in nature; it’s the intellectual part of our brain that’s created the big cities, the demands and the achievements. Our intellect has contributed to the negative spiral without rest; we need the unconditional rest of our natural origins.*”

*Peace and tranquility.* A balanced sensory interaction was experienced as important in order to reach mental peace. The balance was dependent on, e.g., soft colour schemes, familiar plants, things made of natural materials and no interference from alienated interiors (e.g., plastic). The stimuli had to be moderate and not too ordered, so as not to exceed the participants’ mental capacity:

*“It can’t be too chaotic on the walls; the environment has to exude calm, for example there can’t be fire-engine red walls. That wouldn’t have worked; it has to be quite neutral and quite basic. Nothing that disturbs the brain, one rather just...”*

The participants expressed that the total absence of others and the possibility to be alone were very important factors for engaging with nature; the effect of the interaction with nature would be reduced if accompanied by others. This was also expressed as a need to “hide” from others and just be alone with one’s thoughts and feelings. The garden offered many different locations for the participants to seek out: *“… I have wandered down along the trees here, because here you can also be alone and nobody sees you, and one day when I was especially upset and angry too, and didn’t want to be with the group, I wandered down here somewhere… and I guess it’s because in some way it’s about getting as far away as possible and not needing to relate to anybody else, but just being alone.”*

The noise from the highway could sometimes disturb the participants when they were resting in the garden. If this happened, some of them screened the noise with water or by going into a greenhouse or moving to the farthest parts of the garden: “*When I listened to the gurgling water it did away with the traffic noise.*”

Some sounds that were perceived as positive were the birdsong and/or bird twitter, the sound of the wind whispering through the tree canopies and grasses, and the sound of raindrops falling. These were described as being soothing and calming. Repeatedly, participants commented that the sounds of nature are instinctive to us and very familiar since humans have lived in nature for thousands of years, but that the new sounds in the urban context are not as familiar and are therefore more disturbing: “*Natural sounds are more acceptable than industrial sounds and cars; they’re interpreted differently because man has had them around for thousands of years while other sounds are rather disturbing.*”

Close encounters with the natural elements were a source of restoration. Water, in all forms, was described as the source of a deep experience of tranquillity and inner peace; as either snowflakes falling from the sky, the morning fog that looked like a blanket gently covering the garden, or raindrops falling into the pond and making circles on the surface. Many participants told about how they looked at the water’s surface, following raindrops or the fishes in the pond. Participants described that this became a meditative state, a kind of mindfulness that helped them restore their energy: “*When looking in the mirrored surface of the water, I felt how my body was just filled within this calm and was filled with energy. I reach this feeling of meditation where time doesn’t matter.*”

When the participants watched the sky, the clouds and birds helped open their minds and allowed them to dare to see beyond the wall of defense they had mentally built around themselves. The sky had no limits, and one’s mind could be set free without judgment from others. Being one with nature required no words—it was the feeling that mattered: “*But the closer you get to nature the more you end up in some sort of sensuality. Yeah, disconnect parts of the intellect more and more. Like I said, it’s the same as what meditation does; it’s disconnecting the mind, the ego, or whatever you’d call it. Yeah but I mean that it’s nature itself that, I mean, gives something meditative without you having to exert yourself, or if you want to call it something else; but you get that rest in its natural origins without too many intellectual superstructures.*”

Many preferred a secluded place in the sun, feeling the sun on their skin and how the tiredness just ran off them and their whole body relaxed. By hugging a tree or lying on the ground they felt force of nature within, which in turn gave them a feeling of belonging to some greater whole: “*Hugging a tree is delightful and peaceful, it’s a very strong and safe feeling, the tree radiates tranquillity. They (the trees) are strong enough to carry you and protect you. This feels very stable and connects you to some greater whole, a part of a larger life-energy.*”

#### 3.2.2. Awakening and Processing

Two subthemes were distinguished: *Entrusting nature* and *Inspired by nature*. This phase describes the way nature supports and inspires the participants in their process.

*Entrusting nature.* The illness had made the participants feel skinless, exposed and vulnerable, but they expressed that nature embraced them and became a kind of patch on their wounds. The variation of the garden rooms enabled them to seek out a place that harmonized with their moods and needs. Several participants sought support with nature before and/or after attending a session with a therapist: “*…Especially after the Rosen therapy; then I walked down here and kicked things and…then I walked down here, down here is where I walked, and then around, so I could walk and I swore and cursed and cried and carried on all by myself to just be alone and sort of try to let off steam and sort of let out everything X had put into motion.*”

The participants described that nature embraced and supported them when they were processing strong feelings and emotions. Their trust of nature was sincere, and very intimate and deep communication emerged when these often-painful processes came to an end: “*To cry in despair and let go of all the tension that was inside of me; I felt bad and it felt like there was no point living with this. I didn’t want to let go of these feelings in front of other people, but the garden and nature could take it.*”

The possibility to act instantly on the feelings and emotions that were evoked was expressed as crucial in order to move on with the participants’ processes and, most often, bring them to an end. They described that nature helped them “be in their feelings” instead of their head and thoughts, and just let the feelings find their right place: “*When I felt frustration in my body I just wanted to dig something, and I did that for five minutes and then it was over. Before, I had to be at the gym for a long time to get rid of my frustration but now five minutes in the garden is enough and then it’s over.*”

Nature touched the participants on a profound level. Often, when sad or deeply moved, they were in need of a secluded and secure place, out of sight from others, preferably with a view over the surroundings. Some struggled through thorny bushes or sought refuge in primitive and rough places. There was a great need for privacy (the absence of others) to scream and/or cry out loud, growl, throw things, kick and stamp on the ground without someone watching or hearing them. For others, being embedded in vegetation comforted them and enabled them to experience feelings of relief deep within: “*I laid in the grass in the sun, smelled the grass, it felt so good. I laid near the grass and could cry, properly cry like a child; that I had been carrying that for such a very long time.*”

*Inspired by nature*. The participants described the outdoor environment as an infinite world with unlimited freedom, a source of creativity whereby one can discover wonders that evoke delight and joy. Exploring the garden, looking into details and finding new things were experienced as inspiring. There was something enticing about setting out on an excursion to find an unexpected pleasure: “*Walking in the garden offers possibilities to find things you wouldn’t normally see: smiley faces in the pistils, how the insects work and move around. See a mini-world that moves along at its own pace. All this arouses happiness you don’t find at the hospital.*”

As the participants grew stronger they gradually participated in the horticultural occupations, finding them inspiring and joyful. Some participants mentioned that caring for the plants and handling them reduced their stress and allowed a feeling of being happy to emerge: “*So often when I was stressed, I actually went to those geraniums and it made me so happy that they were growing and it made me calm to look at them, and happy that a flower had bloomed and…it just feels so nice.*”

Plants inspired reflection on one’s own needs and life situation, a kind of symbolic communication. For instance, replanting seedlings in larger pots evokes reflection on one’s own situation of “replanting” oneself, needing the right soil and nutrition to grow and prosper in the future. Also, following that little seed as it grew into a larger plant gave some participants hope for their own growth, given the right growth conditions. Some participants mirrored themselves in the plants they found in the garden: “*No, one tree would suppress the other; I didn’t like that…No, I don’t like that—everybody should have space to live, should have a bit of room…it was the symbolism I saw; I mean if it had been somewhere else, but here it absolutely can’t be that way because here everybody’s supposed to get new soil and be able to grow, start again and start to grow, and then there can’t be somebody who sort of suppresses. It was like…yes! Now this tree’s going to sort of rise up, and then I’ll rise up too.*”

### 3.3. Empowerment

The third superordinate theme, *Empowerment*, is characterized as an external process which builds a person’s self-efficacy. One subtheme emerged: *Moving on* including the dimension of *Challenging* *oneself*. In this phase the garden becomes an arena of challenge, where boundaries are broken and new approaches tested before one enters the arena of everyday life with a more sustainable approach to one’s way of living.

#### Moving on

*Challenging oneself.* Many participants described that towards the end of the NBR they possessed an inner strength to be who they are rather than try to live up to an image based on others’ expectations. They were able to face challenges they would have had difficulty encountering before the rehabilitation, and even challenged themselves in different ways to test their newly acquired strengths. Some used the wilder parts to take a challenging walk, finding it a bit fun as well: “*And here it was also that you stumbled over blackberries, so it was a bit of a challenge; I think I’m the type who likes challenges too in some way. I have to go through all the cracks and I have to go through everything there is. I know I was down here a long time and walked where you basically have to crawl and climb, but I’m a bit that way.*”

Many participants described using the outer boundaries of the garden as their breaking point for stepping over their mental boundaries regarding what is and is not acceptable to do. They went beyond the formal boundaries of the rehabilitation garden without knowing whether this was allowed, since the parts beyond contained experimental fields for different crops and an abandoned orchard, closed to visitors. Once they were beyond these boundaries, feelings of freedom and tranquillity emerged. This made them eager to try to break other boundaries in their everyday lives. Some participants used the abandoned orchard to practice “daring” to do forbidden things, e.g., tasting different kinds of apples, throwing away those that were not tasty and seeking out the best ones and enjoying them, or picking the most beautiful flower in the garden or the precious spring flower when hardly anything else alive can be seen, or filling your arms with beautiful pinecones: “*…and that I, like I said, I set up so very many boundaries for myself. I mean I thought Alnarp ended over here, or that rehab ended…Yeah, I’ve said that that’s a high hedge over there so we have to draw the boundary for the area there. I mean it wasn’t until the next to last week that I realized there are apple trees, plum trees. Because I’d put up a certain boundary for the area. I know it still felt like I was playing hooky when I sneaked away for the first time, because you…just this feeling that now I’m doing something wrong. Because I wanted to sneak off, I wanted to have apples. Mm. …and then it ended up that I went there and picked an apple and took a bite, but it tasted bad so I found another one. I tested my way, and sort of, and then I picked a bagful and took it home and a bit like that. Mm. I mean I thought it was really nice to go there and look at the apples and find the right apple and taste it and…take…When I’d done it the first time the way was sort of paved in some way. It’s a funny feeling to be so limited and put up all these boundaries that nobody at all had told me existed, sort of, and then…With a bit of practice, you dare to test things. I think this thing with the apple orchard was really an expression of my sneaking away and wondering if I’m allowed to be here; I actually don’t give a damn if I’m not allowed to be here.*”

## 4. Discussion

The results show that most participants go through three distinct phases during nature-based rehabilitation process. Several aspects of this process can be found in other therapeutic situations [58,59]. What is unique in the NBR is that the participants found support in the natural environments (nature) during all three phases. This partially confirms earlier findings of Grahn and colleagues [24]. However, there are some crucial differences. This applies mainly to the finding of the *Prelude* phase and the component *Social quietness* as a crucial part of NBR. At the end of the discussion, a new theoretical model of NBR is presented.

*Prelude*, this phase describes the safe and secure frame within which the NBR could originate. In this phase, the primary function of the garden was to enhance the feeling of security and offer a world of sanctuary. Perski [16] described three phases of rehabilitation for this client group, the first being the need for a firm and caring attitude from staff as the clients are in a very vulnerable state. In Grahn *et al.* [24], the need for security and the need to make contact with the surroundings are also highlighted. In this situation, it is of utmost importance that both the social and the physical environments are perceived as safe. The team’s role in this phase was crucial for the participants to feel at ease, so that they could let their guard down and not feel alienated.

It seems that the *Prelude* phase was essential in initiating the rehabilitation process, through the establishment of *alliance* and progressing toward the dimension of *permissiveness.* It is recognized that if alliance is not established within the first few sessions there is a profound problem for client and therapist(s) to move further in the rehabilitation process, and it is even recommended that the therapy be terminated [59,60]. Therefore, failure to establish alliance would endanger the progressive move to the next phases of the NBR process and closer encounters with the natural environments*.* Also to be considered is the fact that the participants needed to be in the right phase, *i.e.*, motivated to change and able to participate and receive the treatment offered. Being in the wrong phase could jeopardize their ability to engage from the very beginning [38]. The physical feature of *being away* [29] was experienced as an essential quality that supported the establishment of alliance. The participants expressed that the gateway (the entrance to the garden) marked a distinction between their world of everyday struggle and a world of sanctuary and safe refuge in the rehabilitation garden. Once they passed through the gateway and had closed it behind them, the participants expressed having a strong feeling of being in another world, a world of acceptance and permissiveness. Tenngart Ivarsson [52] also identified the participants’ need to escape from reality (*being away*) and described the garden as a safe place, a refuge from the unwanted distractions of everyday life, a place offering physiological relaxation and psychological contemplation.

During the *Prelude* phase, most occupations were directed towards *just being,* *lowering one’s guard* and *relaxing*, all characterized as *introvert* or outwardly inactive, as from the outside it appeared as if the participants were not doing anything. However, on the inside they were highly activated. The SET recognizes *inward involvement* when a person has a low capacity of executive function, which in this study was the actual state the participants were in when they entered the NBR [24], although SET suggests that participants start to communicate with their surrounding natural environment directly from the start; that the natural environment acts as a therapist from the very beginning. Conversely, results from this study find that for this to happen, participants need a firm *Prelude* phase. Moreover, *Introvert occupations* became important throughout the whole rehabilitation period but diminished towards the end as the participants’ physical and mental capacity grew.

In the *Prelude* phase the team played a more decisive role in accommodating the participants in new circumstances in a new place. In the next phase, *Recuperating*, the role of the garden and natural environments became more distinct as a vital source of support in the participants’ rehabilitation process; here, the process begins to resemble the SET [24].

*Recuperating*—Korpela and Staats [42] argue that by being alone with nature one can restore oneself from both emotional and cognitive stress, and that the company of others may degrade this restoration due to interference with one’s engagement with the natural environment. The subtheme *Restoration* was characterized by the participants’ rest and recovery in the tranquil natural environment—*i.e.*, alone. The participants specifically expressed that the total absence of others, in this study defined as *social quietness*, was as an important factor for engaging with nature and reaching inner peace and tranquility. This has to do with both sounds and noise from other people as well as the actual presence of another person in one’s surroundings. The engagement with nature was negatively affected when others physically entered the scene. The participants described how nature had an all-embracing role when it was not shared with others. This concurs with Ottosson’s [51] own experience of solitude in nature. The natural setting became a vital source of healing and recovery when free of other people, who would disturb his interaction and communication with nature.

The participants frequently mentioned the soothing sounds of nature, particularly birdsong and/or bird twitter, as calming and joyful. Recent studies found that the chances of stress recovery were higher when sounds of nature were present, particularly birdsong [61,62]. Grahn and Stigsdotter’s [63] findings also suggest that the calming sounds of nature were associated with stress recovery in an urban context. These sounds are the properties of the dimension *Serene* [63] as well as of the absence of disturbing noise or disturbing people, and bear a resemblance to the suggested term *social quietness*.

**Figure 2:**
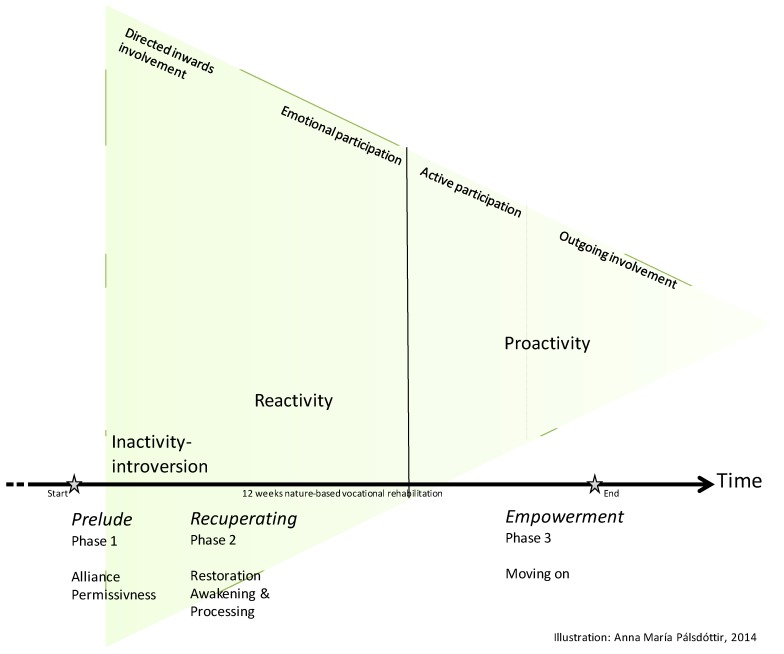
An explanatory model of nature-based rehabilitation that illustrates the three phases of rehabilitation—*Prelude*, *Recuperating* and *Empowerment*—over time, the occupational repertoire (introvert, reactive, and proactive occupations or *IRP occupations*) and the SET pyramid of executive functions. The *IRP occupations* are connected to the different phases of the rehabilitation process, and correspond to the suggested levels of executive functions in the SET pyramid.

Nature’s great capacity to offer opportunities for effortless *quiet fascination* seemed to become a source of restoration, as Kaplan *et al.* [64] suggest, and the intimate communications involved all senses, deepening the experience as Grahn *et al.* [24] suggested. This also resembles Ottosson’s [51] own experiences, whereby he seeks support from the surrounding environment in the process of a journey of recovery after experiencing severe brain damage due to a traffic accident. He found this support in a nearby forest, seeking the comfort of stones, water and plants in his difficult time of recovery. The subtheme *Restoration* bears a resemblance to the lower part of the SET pyramid in regard to the participants’ executive functions and inward involvement [24]; see Figure 2.

In the subtheme *Awakening & processing*, the garden was entrusted with both sad and happy emotions. The participants sought out secure places, out of sight from others but with an outlook over the surrounding area so they would not be taken by surprise, where they, alone, could react based on their needs. These places resemble the dimensions *Refuge* (“*A sanctuary, a safe enclosed place… where you can relax and be yourself, and also experiment and play*”) [24] and *Compatibility* (fitting with and supporting what one wants or is inclined to do) [29]. Both these characteristics are regarded as properties of restorative and supportive environments.

The participants expressed that the possibility to instantly act on the emotions evoked was crucial in order to move on with the recovery processes, and not to be left with them in their heads. This, the participants often repeated, was not possible in the *usual* health care facilities. Other studies have also recognized the added value of having nature to turn to during the recovery process and nature being an essential factor for handling the ongoing processes [38,40,52,65]. Before going to a therapy session, many participants prepared themselves by walking or sitting in the garden, mentally preparing for what was coming. Already in 400 BC, Hippocrates instructed his clients to prepare themselves for an operation through a calm walk in nature, and also recommended this as a vital part of their recovery afterwards. Hippocrates claimed that the doctor should rely primarily on nature and man’s own healing powers, reflecting a salutogenic view on recovery [66,67].

From an occupational perspective, during *Awakening* & *Processing*, the later phase of *Recuperating*, the participants began to perform *reactive* occupations, which thus became increasingly dominant over the introvert occupations. Reactive occupations could be characterized as a response to external stimuli, e.g., therapeutic conversations and actions in the NBR, awakening demanding inner feelings, or other participants’ doing and being, which often mirrored their own actions they had not previously reflected on. Also, the natural environment initiated reactive occupations, mainly having to do with a participant’s reflections on his/her own life. This can also be illustrated, as is done in the SET pyramid of executive functions, as *emotional* to *active participations* (see Figure 1) [24].

The last subordinate theme, *Empowerment*, handles the phase in which most of the participants *moved on* by *challenging themselves*. Empowerment is characterised by an external process which builds a person’s self-efficacy self-esteem and confidence in his/her ability to make good decisions, to control his/her own life and to achieve autonomy [68]. The garden thus became an arena where they could try things out and break their perceived boundaries—which held them back from living a good life according to their own needs and then transfer this to everyday life situations. This is in line with the findings by Eriksson *et al.* [40] whereby the social supportive environment was recognized as an important bridge of experiences from the NBR to everyday life. This is also recognized to be a “try out-lab” for improving everyday function and well-being [65].

The current findings concerning the supportive environment at the Alnarp Rehabilitation Garden resemble the properties of places where one can act according to one’s own needs and desires; *i.e.*, the *Refuge* dimension [24] and the property of *Compatibility* [29]. Both of these are components of a restorative environment, and *Refuge* is an important component of a supportive environment [24].

From the occupational perspective; *proactive occupations* came to dominate in this last phase, as the participants challenged themselves based on their own needs and desires by taking initiatives to occupy themselves and explore the outer borders of the garden, illustrated in the SET pyramid of executive functions as *outgoing involvement* (see Figure 1) [24].

*An explanatory model that illustrates the nature-based rehabilitation at Alnarp.* Figure 2 illustrates the three phases of rehabilitation—*Prelude*, *Recuperating* and *Empowerment*—over time, the occupational repertoire (introvert, reactive and proactive occupations or *IRP occupations*) and the SET pyramid of executive functions. The *IRP occupations* are connected to the different phases of the rehabilitation process, and correspond to the suggested levels of executive functions in the SET pyramid. For instance, in the *Prelude* phase the introvert occupations dominate the occupational repertoire, resembling direct inward involvement in the SET pyramid. In the *Recuperating* phase the reactive occupations take over, resembling the emotional participation moving towards active participation. Finally, in the last phase, *Empowerment*, the proactive occupations dominate, resembling the outgoing involvement of the SET pyramid. The lengths of the phases vary individually, but are illustrated in the sequence in which the participants described their occurrence. This is an effort to illustrate how nature and nature-based occupations can facilitate and support the rehabilitation process.

In this study, the natural environments seemed to possess a great capacity to support and embrace the participants’ rehabilitation processes as they were being evoked by the professional rehabilitation team. It would be of interest to identify specific locations in the rehabilitation garden at Alnarp that were experienced as supportive, and the qualities of these locations. Based on this, we would like to see further studies investigating and describing the physical features by which the natural environments can affect and support the rehabilitation process for this patient group.

## 5. Methodological Considerations

This article is based on interviews with 43 participants representing the patient group regarding, e.g., symptoms as well as distribution in age and gender. Data were collected for four years, involving different seasons weather and group constellations. Yet, similar processes recurred and similar dynamics emerged. Many participants had very little experience working in a garden, or even being in natural areas, while others had more experience. This is likely representative of the patient population.

Several participants wanted to show gratitude for having had the opportunity to participate in the NBR, but all had both positive feedback as well as more critical comments. They were overwhelmingly positive to the possibility to be outdoors in “nature” in rehabilitation, but made critical comments about the scheduling of some activities and the traffic noise from the highway, which could be stressful in certain weather conditions (which made the noise worse).

There are some limitations of generalization as the NBR context may vary in terms of rehabilitation team, group size and the physical location. Further, no comparisons have been done with other types of rehabilitation for this group of participants and therefore difficult to generalize about the importance of access to natural environments when undergoing rehabilitation for stress-related mental illnesses.

Considering the large amount of data and the comprehensive analysis work (1190 h) the first author had the overall responsibility for the analysis process. The process of analysis was conducted by first author and was closely followed throughout by the last author to ensure the trustworthiness of the analysis process.

## 6. Conclusions

For most participants, the NBR process included three main phases (*Prelude*, *Recuperating* and *Empowerment*) supported by natural environments, rehabilitation team and other participants (see Figure 2). The profound non-verbal communication with nature, in the garden, seems not only to be a source of restoration but also to have reconciled complex mental processes during the NBR. Some characteristic components of the supportive environment were identified: *Being away*, *Compatibilit*y, *Serene*, and *Refuge*. This, to a certain extent, concurs with the original design concept of the Alnarp rehabilitation.

The *Prelude* phase seems to be the key for the phases that follow, *Recuperating* and *Empowerment,* to take place. Further, *IRP occupations* are a new way of describing the types of occupations that are important in NBR for individuals with related illnesses. An explanatory model of “NBR” is presented, including the three phases of NBR, *IRP occupations* and a SET pyramid of executive functions (Figure 2). Also, a new component of restorative environment was identified, *i.e.*, *Social quietness*, as an important component facilitating personal and intimate engagement with the natural environments in the *Recuperating* and *Empowerment* phases.
